# The Role of Hub Neurons in Modulating Cortical Dynamics

**DOI:** 10.3389/fncir.2021.718270

**Published:** 2021-09-24

**Authors:** Eyal Gal, Oren Amsalem, Alon Schindel, Michael London, Felix Schürmann, Henry Markram, Idan Segev

**Affiliations:** ^1^The Edmond & Lily Safra Center for Brain Sciences, The Hebrew University of Jerusalem, Jerusalem, Israel; ^2^Department of Neurobiology, The Hebrew University of Jerusalem, Jerusalem, Israel; ^3^Blue Brain Project, École Polytechnique Fédérale de Lausanne (EPFL), Campus Biotech, Geneva, Switzerland

**Keywords:** network science, cortical microcircuitry, network synchronicity, scale free network, hub neurons, small world topology, cell ablation strategy

## Abstract

Many neurodegenerative diseases are associated with the death of specific neuron types in particular brain regions. What makes the death of specific neuron types particularly harmful for the integrity and dynamics of the respective network is not well understood. To start addressing this question we used the most up-to-date biologically realistic dense neocortical microcircuit (NMC) of the rodent, which has reconstructed a volume of 0.3 mm^3^ and containing 31,000 neurons, ∼37 million synapses, and 55 morphological cell types arranged in six cortical layers. Using modern network science tools, we identified hub neurons in the NMC, that are connected synaptically to a large number of their neighbors and systematically examined the impact of abolishing these cells. In general, the structural integrity of the network is robust to cells’ attack; yet, attacking hub neurons strongly impacted the small-world topology of the network, whereas similar attacks on random neurons have a negligible effect. Such hub-specific attacks are also impactful on the network dynamics, both when the network is at its spontaneous synchronous state and when it was presented with synchronized thalamo-cortical visual-like input. We found that attacking layer 5 hub neurons is most harmful to the structural and functional integrity of the NMC. The significance of our results for understanding the role of specific neuron types and cortical layers for disease manifestation is discussed.

## Introduction

Research at the macro- and peso-scale brain anatomy has demonstrated a clear connection between structure and function. Indeed, the global network structure of the brain was shown to be altered in diseases such as schizophrenia (Rubinov and Bullmore, 2013), bipolar disorder (Syan et al., 2018) and others (Stam, 2014). Yet, pathology takes place at the micro-scale, at the cellular and synaptic level architecture of neuronal microcircuits. How the connectomics at this level shape the dynamics and functionality of biological circuits is indeed a key question in neuroscience (Abbott et al., 2020; Amsalem et al., 2020; Turner et al., 2020). Of particular interest is the impact of structural disruption of the connectome, whether due to natural aging or due to disease. These two types of disruption are rather different. Whereas a non-selective general reduction in the number of cells was found in the aging brain, recent studies showed selective cell vulnerability associated with certain pathologies. For example, a significant decrease in the number of specific cell types in cortical areas were found in Alzheimer’s disease (Stranahan and Mattson, 2010; Fu et al., 2018; Murray et al., 2018), multiple sclerosis (Schirmer et al., 2019) and Parkinson’s disease (Hammond et al., 2007).

Experimental investigation of the role of specific cell populations in the neocortex has advanced significantly in recent years. Optogenetic methods (Deisseroth, 2015) together with genetic dissection of specific neurons (Luo et al., 2018) enables precise recording and manipulation (silencing and activating) of specific neuronal populations. Manipulating the neural activity of specific cell types during *in vivo* experiments is presently used to affect animal behavior (Guo et al., 2015; Carrillo-Reid et al., 2019; Robinson et al., 2020), but the effects of such cell-type-specific manipulations on circuit dynamics are rarely characterized at the network scale (Cardin et al., 2009; Pouille et al., 2009; Adesnik and Scanziani, 2010; Xue et al., 2014; Bitzenhofer et al., 2017). Consequently, we currently lack understanding of the role of particular cell populations, e.g., in the case of specific diseases, in shaping neural network dynamics and eventually network functionality.

To address this gap, we hereby utilized network theoretical approaches to explore the correlates between microcircuitry structure and function, as theoretical approaches provided new insights into the relationship between structure and function of neural networks (Newman, 2003; Avena-Koenigsberger et al., 2017; Nolte et al., 2020). Toward this end we simulated the most up-to-date, biologically realistic, dense digital reconstruction of a neocortical microcircuit, NMC (Markram et al., 2015). This 0.3 mm^3^ cortical circuit contains some 31,000 neurons, ∼37 million excitatory and inhibitory synapses, and 55 morphological cell types (m-types). This model circuit enables an unprecedented opportunity to directly investigate the impact of network structure on system dynamics by introducing cell-specific and layer-specific attack/damage while measuring the collective neural activity under different physiological conditions.

This cortical microcircuit displays structured architecture with multiple emerging network features at multiple levels, from the local single-cell level, through clusters and motifs of two and three neurons, to the global network-wide architectures (Gal et al., 2017). In particular, at the single-cell level, the circuit contained highly connected neurons (“hub neurons”) that either received or made significantly more connections than average. Moreover, these hub neurons belong to a surprisingly small number of cell-type subclasses and are densely interconnected among themselves forming a cell-type-specific core of hubs (“rich club”). Among all 55 m-types that constitute the mouse somatosensory cortex, the hubs belonged mainly to pyramidal cells from the deeper layers 4–6. Hub neurons with a high number of outgoing connections (“out hubs”) tend to arise from the intermediate layers (4–5), and those with a high number of incoming connections (“in hubs”) were positioned more deeply (in layers 5–6). Surprisingly, the existence of such cell-type-specific wiring specificities was found to be essentially unavoidable, emerging mostly from the asymmetrical structure of individual cortical neurons (Gal et al., 2019). However, determining the functional role of such ubiquitous wiring specificities in shaping the cortical circuit dynamics remains elusive (Setareh et al., 2017; Luccioli et al., 2018). Indeed, the question emerges: is the death (“attack”) of these hub cells, or of specific cell types, in realistic cortical microcircuits more harmful to the network dynamics as compared to that of other cell types?

Using network science tools, we first analyzed how the circuit structural connectivity was impacted after removing these cells. At the global level, the structural connectivity demonstrated small-world topology with an average synaptic distance of 2.5 synapses separating any two neurons. In the present study, we measured the increase in the average synaptic distance (mean shortest path in a network; see section “Materials and Methods”) before and after hub attacks; this measure is related to the efficiency of information flow across the network (Watts and Strogatz, 1998). Another respective measure of the network topology is the clustering coefficient, which measures by the tendency of nodes to cluster together (see section “Materials and Methods”). When a network simultaneously displays a short-averaged path length and a high clustering level it is termed as a “small-world network” (Watts and Strogatz, 1998). Reduction in the “small-worldness” of the networks might imply a reduction in efficiency of information exchange and capacity for associative memory (Bullmore and Sporns, 2009).

We next used several measures to evaluate network dynamics such as mean firing rate, coefficient of variation (CV), SPIKE-synchronization (Kreuz et al., 2015), etc. Each measure provides us with different aspects of the circuit activity and, hence, helps us understand how targeted attacks on hub neurons are more disruptive to the network functionality than attacking the same number of neurons randomly. Combining these results with structural network measures following cells’ attack sheds new light on the robustness of the NMC to a variety of attacks and, at the same time, on the functional sensitivity of the network to some of these attacks. These findings provided important insights into the impact of the death of specific cell types (e.g., due to certain diseases) on the dynamics and functionality of local cortical microcircuits.

## Results

### Hub Neuron Attacks Impact the NMC Small-World Topology

To explore the structural and functional impact of attacking highly-connected hub neurons we started by ranking all neurons according to their total degree (total number of pre- and post-synaptic cells connected to a given neuron). We then removed (attacked) different quantities of these neurons, starting from the highest degree hub cells to the lowest degree.

**FIGURE 1:**
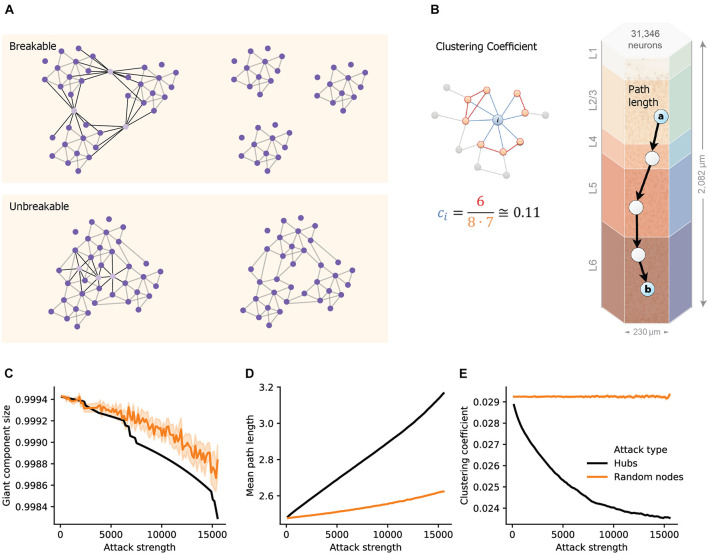
Structural disruption of cortical microcircuits following attacks on hub neurons. **(A)** Schematic illustration of two network architectures having different sensitivity to a similar targeted hubs attack. (Top) The case of breakable network whereby an attack on 3 hub neurons disintegrates the network into 3 separate sub-networks. (Bottom) The case of an unbreakable network which, after an attack on 3 hub neurons, remains fully connected. **(B)** Schematic illustration of the two features used to define small-world networks. (Right) The shortest path length connecting two nodes (cells “a” and “b”, overplayed on the modeled neocortical microcircuit). (Left) The local clustering coefficient of a node *i*, *c***_*i*_**, which is the density of connections among the neighbors of this node. In the example shown, node *i*, has 8 neighbors; among them only 6 connections out of all 8⋅(8–1) = 56 possible connections (*c*_*i*_ = 6/56 ≅ 0.11). **(C)** The size of the giant component in the NMC as a function of the number of hubs attacked (Attack strength), black line, compared to the corresponding random attacks (orange line), demonstrating that the NMC network is “unbreakable.” **(D)** Mean path-length and **(E)** mean clustering coefficient following hub attacks (black) versus random attack (orange). These two features are particularly sensitive to hub attack. Light orange depicts 95% confidence interval in all figures.

To quantify the structural effect, we first measured the overall connectedness of the network, as captured by the size of the network’s largest connected component (giant component size; see section “Materials and Methods”). We highlight two extreme outcomes on network architecture following hubs attack (Figure 1A). On one extreme, the removal of hub neurons may completely break the network into multiple smaller unconnected components (Figure 1A top; “breakable”). On the other extreme, the removal of hub neurons will not break the network and the remaining neurons will remain in one connected giant component (Figure 1A bottom; “unbreakable”). A finer structural feature utilized here, which relates to the efficiency of network communication and computation, is the small-world topology of the network. This measure relies on two opposing requirements: a short path length between any pair of nodes/neurons (Figure 1B, right; see section “Materials and Methods”) and clustered interconnectivity, *c*, within groups of nodes (Figure 1B, left; see section “Materials and Methods”). Thus, the “small-worldness” of a neural network reflects the degree in which it balances the needs for global integration and local segregation of neural information (Sporns, 2013a).

We found that the giant component of the NMC is not broken by hub-attack as its size only negligibly decreased when a large number of hub neurons are attacked (Figure 1C, black line; see section “Materials and Methods”), showing that the global integrity of the circuit is robust to such attacks. Moreover, the effect was similar to respective random cell attacks (Figure 1C, orange line) in which a similar number of cells were selected randomly (see section “Materials and Methods”). This implies that the NMC circuit is of the unbreakable type. In contrast, the small-world topology of the circuit is more sensitive to attacks on hub neurons. For example, attacking 5,000 hub neurons increased the path length from 2.48 to 2.69 (8% increase, Figure 1D) while reducing *c* from 0.029 to 0.025 (12% reduction, Figure 1E). To test whether this disrupted small-world topology is expected by chance and resulting merely due to the number of eliminated nodes, we performed control random attacks with matching number of nodes (Figures 1C–E). We found that the disruption of both path-length and clustering due to hub attacks were significantly stronger than that expected from the random attacks (*p* < 0.001 for both, two-tailed Wilcoxon rank sum test; *n*_1_ = 10, *n*_2_ = 100; see section “Materials and Methods”). Additionally, the observed disruptions were found significant (*P* < 0.001, two-tailed Wilcoxon rank sum test; *n*_1_ = 10, *n*_2_ = 100; see section “Materials and Methods”) compared to that of randomly attacked networks with similar numbers of eliminated edges (Supplementary Figure 1).

### Hub Neurons Are Key for Network Synchrony

The structural analysis has uncovered the disruptive effect of hub attacks on the small-world properties of the neocortical microcircuit. However, the functional implications of such structural changes are not trivial. To elucidate the functional impact of hub neurons on network dynamics, we simulated NMC networks following different cells’ attacks and examined various functional features of network activity.

**FIGURE 2:**
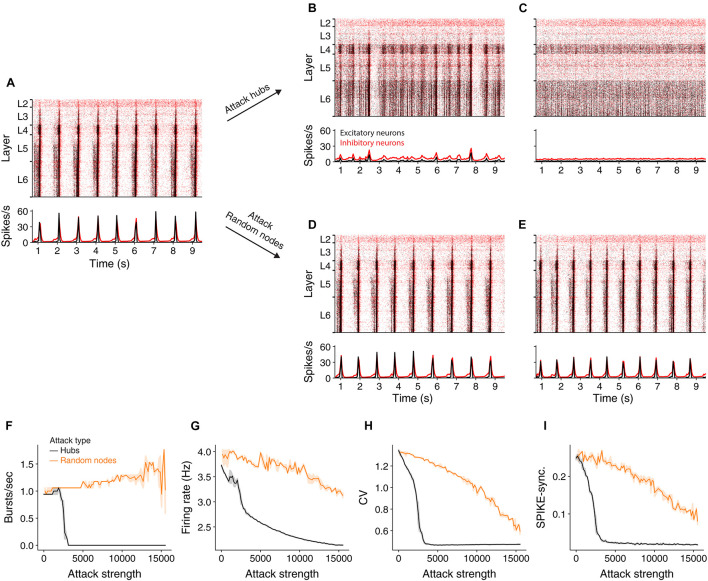
Effect of hub attacks on the dynamics of the NMC network in the synchronous regime. **(A)** Raster plot (top) and time histogram (bottom) of the NMC spiking activity during spontaneous bursting state (see section “Materials and Methods”). In this state, all cortical layers tend to burst synchronously at about 1 Hz, with lower layers starting to fire earlier. **(B,C)** Same as **(A)** after attacking 2,977 and 7,993 hub neurons, respectively. **(D,E)** As in **(B,C)** but for respective random attacks. **(F,G)** Impact of hubs attack (black) versus random attack (orange) on network activity as a function of attack strength. **(F)** Impact on the number of bursts/sec. **(G)** Impact on average firing rate. **(H)** The impact on the coefficient of variation. **(I)** On global SPIKE synchronization measure. For all measures, hub attack is significantly more impactful (light orange depicts 95% confidence interval).

It has been shown (Markram et al., 2015) that at an extracellular calcium concentration of 1.4 mM the NMC network generates spontaneously synchronous bursts at ∼1 Hz (Figure 2A and see section “Materials and Methods”). At this synchronous state, the cells’ mean firing rate is 3.7 Hz, their CV is 2.16 and SPIKE-synchronization measure is 0.25 (see section “Materials and Methods”). We simulated the circuit after removing 2,977 hub neurons (Figure 2B) or 2,977 random neurons (Figure 2D). Removal of hub neurons markedly reduced the number of bursts in the network (Figure 2B), whereas the bursting properties of the network were unaltered for the respective random attack (Figure 2D). Increasing further the number of attacked hub neurons to 7,993 completely abolished the bursting activity of the network, shifting it to the asynchronous state (Figure 2C) whereas it did not change the burstiness in the case of random cells removal (Figure 2E). Figure 2F summarizes the change in burst number due to different strengths of attacks. In addition to the reduction in burst activity due to removal of hub cells from the circuit, the coefficient of variation (std divided by mean ISI), CV, firing rate and spike-synchronization all showed a stronger reduction compared to removal of random neurons (Figures 2G–I).

Because hub neurons are mostly excitatory (Gal et al., 2017), hub attacks primarily remove excitatory neurons from the network. Indeed, in all analyzed hub attacks, ranging up to 15,000 neurons, the percentage of excitatory neurons in the attacked neurons was above 97%, but when attacking random nodes, we converge to the full circuit distribution of E/I neurons (85% excitatory). To address this discrepancy, we performed random attacks that matched both the number and the E/I identity of nodes (Supplementary Figure 2 and see section “Materials and Methods”). Indeed, this attack was more disruptive than the completely random attacks, but still less than the hub attacks. We also show that the number of edges attacked is not the main factor for this effect (Supplementary Figure 2). This analysis demonstrates that, in the synchronous state, the impact of a neuron on the generations of collective synchrony in the NMC is more affected by their embedding in the network (“hubiness”) rather than by their physiological effect (the network E/I distribution).

Finally, we repeated the above analysis also for the asynchronous state, which is induced by setting the calcium concentration in the NMC simulations to 1.25 mM (Markram et al., 2015). At this state, the difference between attacking hubs versus random neurons is still significant, but not prominent as in the asynchronous case (Supplementary Figure 3; see section “Materials and Methods”). Indeed, the asynchronous state is, in general, more robust to cell-attacks.

### Functional Implication of Layer-Specific Hub Attacks

**FIGURE 3:**
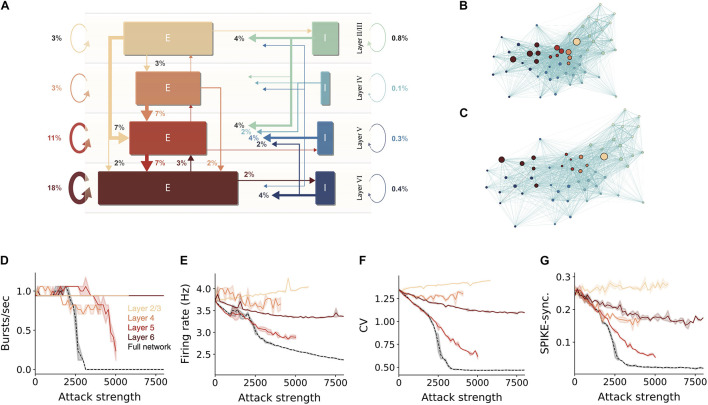
Sensitivity of network dynamic to hub attack is mostly attributed to attacks on L5 PCs. **(A)** Percentages of synapses for excitatory (brown-red arrows) and inhibitory (blue-green arrows) connections in the NMC microcircuit (layer 1 omitted). Arrow width and corresponding numbers indicate the percentage of total synapses formed by this pathway (omitted for pathways with <1% of synapses). The total percentage of plotted synapses is 98%; the remaining 2% originate in layer 1. Rectangle sizes are proportional to the sizes of the corresponding number of excitatory or inhibitory populations. **(B)** The connectivity among layers and cell-types is visualized using the force-directed graph algorithm (see section “Materials and Methods”). The network is composed of 55 morphological cell-types (circular nodes, colors match to that in **A**); edges strength corresponds to pairwise connection probabilities. Strongly connected cell-types are displayed closer in space. **(C)** Same as **(B)**, but for the network following an attack on 15,000 hub neurons. Note the disappearance of the large nodes in Layer 5. **(D–G)** Different quantification of network activity for layer-specific hub attack, global hub attack and random nodes attack. **(D)** Number of bursts/sec, **(E)** average network firing rate, **(F)** coefficient of variation, **(G)** global SPIKE synchronization measure (see section “Materials and Methods”). Note that attack of L5 pyramidal cells is the most disruptive layer attack.

We showed that hub neurons are more effective in driving network synchrony. These hubs potentially belong to multiple cell-types at the different layers. To further detail the impact layer-specific hubs, we measured the connectivity among excitatory and inhibitory cells within and between layers (Figure 3A). To compactly examine the connectivity among all 55 cell types in the NMC, we employed a force-directed graph drawing algorithm, whose 55 nodes depict the cell types whereas edge strengths correspond to the pairwise connection probability. In this presentation, tightly connected nodes will tend to appear closer. Inspecting the original network, the existence of large cell-type groups and clusters can be seen within each layer (Figure 3B large nodes). After attacking 15,000 hub neurons, several changes were prominent (Figure 3C). The network layout had spread more widely, indicating that the strength of the connections between cell types is reduced. Additionally, the large nodes of L5 almost disappeared, hinting to their possible impact on disruption of network functionality (see below).

To test the importance of the different layers for network activity we performed layer-specific hub attacks by simulating the circuit while removing hub neurons in specific layers. We found that, in general, attacking L5 hub neurons is the most disruptive attack as it caused the largest change in all the functional measures used (Figures 3D,F,G, but see the impact of L4 in Figure 3E). When revisiting the high-level circuit connectivity (Figure 3A) one observes that L5 excitatory cells are the most interconnected population in the circuit per cell (the overall percentage of incoming and outgoing connections); this is probably the reason for the high functional influence of L5 attack. L6 attacks also resulted in a large change of the functional measurements except for the number of bursts; similarly, L6 excitatory cells are also relatively highly interconnected (Figure 3A). We also note that when attacking L2/3 there is a slight increase in the network firing rate, this might be due to the relatively large part of inhibitory neurons in this layer.

We summarize this section by noting that attacking hub neurons is most disruptive to network dynamics when hub neurons are attacked at all layers (Figures 3D–G, dashed black line). Layer 5 is the most-sensitive layer to such an attack. As neurons in different cortical layers belong to different genetic types (Gouwens et al., 2019; Yuste et al., 2020), this result shows that, although the same number of neurons might be degraded due to different pathologies that target specific genetic cell types (e.g., L5 thick-tufted pyramidal cells), they will have a very different impact on the overall dynamics of the cortical network and, thus, on the manifestation of specific diseases.

### Functional Implication of Hub Cells on Thalamic Input Processing

**FIGURE 4:**
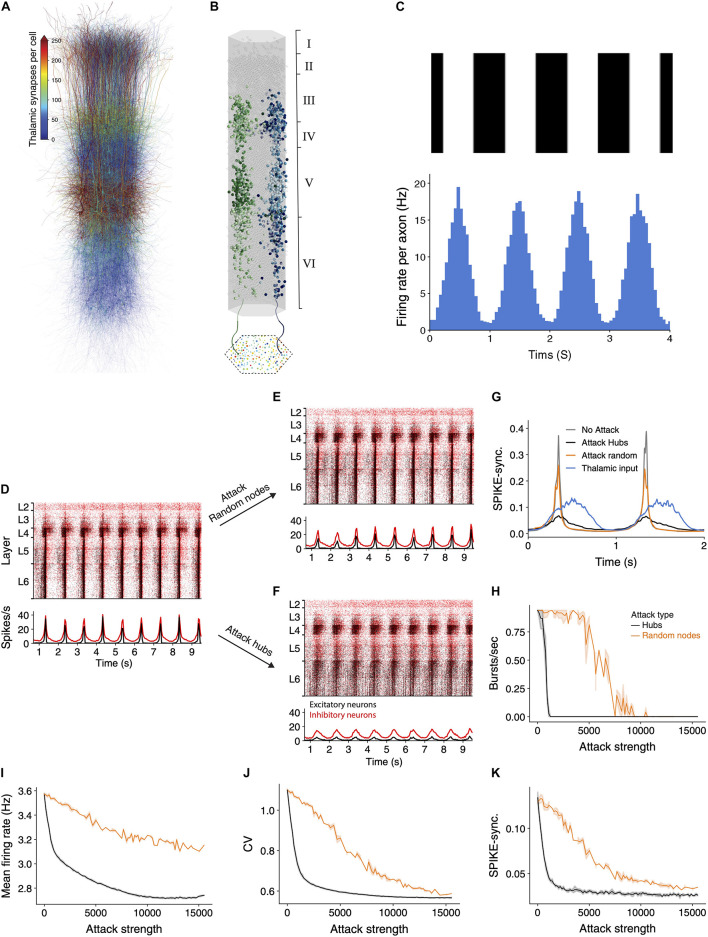
Network synchrony due to thalamic input is disrupted following attack on hub neurons. **(A)** Schematic illustration of the NMC circuit, each neuron is color-coded by the number of thalamic synapses it receives. **(B)** The spatial distribution of the thalamic input is illustrated by showing the cortical postsynaptic neurons receiving inputs from two exemplar TC axons (green and blue axons and respective colored cortical neurons). **(C)** Mean thalamic activity **(bottom)** simulating their response moving bars at 1 Hz (**top**, see section “Materials and Methods”). Bin size = 45 ms. **(D)** Raster plots of the circuit responding to the thalamic input. This circuit fires asynchronously at its spontaneous state ([Ca^2+^]_0_ = 1.25 mM; see Supplementary Figure 3A and section “Materials and Methods”). Red, inhibitory neurons; black, excitatory neurons. **(E,F)** The response of the circuit to the thalamic input after attack of 2,500 random nodes **(E)** and 2,500 hub neurons **(F)**. **(G)** Circuit’s SPIKE-synchronization profile (Kreuz et al., 2015) in response to thalamic input is sharpened in the intact and randomly-attacked networks as compared to that of the TC input itself (blue line) and it decreased dramatically after hub attacks (black line), even below that of the TC input. **(H–K)** Different quantifications of network activity under thalamic input for hub and random attacks (as in Figures 2F,G).

The above sections have demonstrated that hub attacks are significantly more effective in disrupting circuit-wide synchronization in the spontaneous synchronized case. In this section, we set to test whether this observation is general enough, and also valid for the case where the synchronized activity is generated by realistic sensory input. Toward this end we innervated the NMC circuit by 574 thalamic fibers (the thalamo-cortical, TC, input). These TC axons project mostly to neurons in lower layers 3 and 5 (Figure 4A), where some neurons might receive up to 750 thalamic synapses. Each reconstructed axon is making synapses on dendrites that are adjacent to its path (Figure 4B; see section “Materials and Methods”), functionally impacting a vertically confined space (Amsalem et al., 2020).

For these simulations we set the calcium concentration value to 1.25 mM; this results in the network being in a spontaneous asynchronous state (as in Supplementary Figure 3A; see also **Figure 15** and **Supplementary Figure 12** in Markram et al., 2015). We then simulated a grating drifting at 1 Hz by generating the firing rate of the thalamic axons from an inhomogeneous Poisson process with a time-varying rate that followed a sinusoidal function (Figure 4C; see section “Materials and Methods”). The circuit responded by following the oscillatory input firing with highly time-locked synchronized bursts of spikes at 1 Hz (Figure 4D). We repeated the simulation following the attack on 2,500 random neurons (Figure 4E) or 2,500 hub neurons (Figure 4F). In the random attack the circuit continued to follow the oscillatory input and the response remained synchronized to the input. However, following attack on hub neurons, the circuit response was much less synchronized.

We next quantified the circuit activity using SPIKE-synchronization time profile in response to the thalamic input for different cases (Figure 4G). The blue line in this figure shows the spike-synchronization measure of the TC axons whereas the gray line depicts the spike-synchronization of the cortical neurons. We found that the circuit strongly sharpens the synchronicity of the thalamic input (Figure 4G, compare gray to blue line), and that attacking random nodes (in this case 2,500) only slightly reduced this sharpening (orange line). In contrast, attacking (2,500) hub neurons reduced the synchronization in response to the TC input dramatically (Figure 4G, black line); this case is even less synchronized than the thalamic input itself (Figure 4G, compare black line to blue line). We further conducted a complete set of simulations while attacking random or hubs neurons, and quantified different functional features (Figures 4H–K). Hub attacks were much more destructive compared to the random attacks and caused a larger change of all measures. Interestingly removing hub neurons reduced the SPIKE-synchronization profile to a value which is lower than that of the input, showing the strong dependence of the circuit ability to follow and sharpen synchronized input on hub neurons. These results highlight the importance of hub neurons in processing sensory input, clearly demonstrating that the integrity of the cortical hub neurons is critical for the fast and reliable response of the cortical circuit to sensory information.

## Discussion

We introduced in this work a network-based approach to investigate the relation between a cortical circuit structure to its function by removing cells according to different criteria in a highly detailed simulation. Additionally, we proposed a network-based approach for identifying potentially interesting neurons. Our analysis shows that the importance of a neuron in maintaining synchronous activity is more affected by their embedding in the network (e.g., the neurons’ in/out degree, its “hubness”) rather than strictly by their physiology.

To examine the importance of different cells and layers, we simulated the network activity after removing hub cells globally or from specific layers and compared the results to control models where random cells were removed. We discovered that hub neuron attacks have the largest change of structural network measures, leading to loss of the small-world properties of the simulated NMC (Figures 1C–E). Accordingly, attacks on this population resulted in the largest decrease in the network synchrony, firing rate and number of bursts (Figure 2). The attack changed the network response from spontaneously synchronous to asynchronous, resulting in no bursts and reduced CV, as noted by another recent research (Nolte et al., 2019).

Among attacks targeting specific layers, mimicking a more biologically plausible scenario, we found that attacking L5 hub neurons resulted in the largest effect on all functional measures (Figures 3D–G) and therefore is the most distributive attack. We believe that this phenomenon is rooted in the high interconnectivity of L5 excitatory neurons (Figure 3A). Interestingly the specific genetic profile of L5 excitatory neurons is widely used to optogenetically target and record from this subset of neurons (de Vries et al., 2020), and open the possibility of examining our predictions by specifically silencing this subpopulation while recording from the neocortex.

Hub neurons play an important role not only in maintaining spontaneous network oscillatory activity but also in the processing of sensory input from other brain areas. When thalamic (sensory) drifting sinusoidal input impinged on our modeled cortical circuit, the circuit not only followed the thalamic synchrony, but resulted in activity that was more synchronized. We then found that attacking hub cells caused a significant reduction of the synchrony of the cortical column with respect to the oscillation of the TC input (Figures 4G–K), eventually resulting in activity that is less synchronized than the input. Nevertheless, random attacks seem to have a minor effect on thalamic input processing by the network, demonstrating yet again the robustness of the cortical microcircuit to random cell death. These results highlight the functional role of hub neurons in fast processing of sensory information in the cortical microcircuit.

In a previous study we have demonstrated that both in-hubs and out-hubs neurons belong to a small subset of cortical neuron types (Gal et al., 2017; Figures 3A,B). It was recently shown that, although any disease-associated genes are expressed in multiple cell types, the pathologic variants affect primarily specific cell types (Guan et al., 2021), and see also related recent work of disease map and specific cell type in the retina by Siegert et al. (2012). Therefore, it is highly likely that dysfunction of specific genes might abolish specific hub cells. We further emphasize that hub neurons (both in-hubs and out-hubs) require more metabolic energy as they receive/form a larger number of synapses and this might entail earlier death (e.g., in neurodegenerative diseases) compared to non-hub neurons (see e.g., Pathak et al., 2013).

Our findings can also be seen as a demonstration of how network science theory is implemented in realistic networks. As the modeled cortical microcircuit was shown to display small-world topology with a high clustering coefficient and short mean path, we now can systematically characterize how the targeting of fundamental components of the network, the hubs, indeed leads to loss of this topology in the neural microcircuit, and results in major functional disruptions. Robustness of complex networks to random attacks and vulnerability to removal of highly connected nodes was previously shown theoretically (Watts and Strogatz, 1998; Boccaletti et al., 2006). Here, we provided concrete evidence that hubs fulfill their theoretical key role in affecting the dynamics of the network, in a highly detailed biological model circuit. The loss of small-world topology among brain regions was shown to be related to neurodegenerative diseases using fMRI data (Sporns, 2013b). Our theoretical experiments suggest that a decrease in network clustering and an increase in the mean short path length can cause functional failures also at the microscale level of resolution.

To conclude, while neurons are usually characterized according to genetic markers, morphology and physiology (Berg et al., 2020; Yuste et al., 2020), we showed how a specific structural measure such as the number of synapses that defines hub cells, has a direct effect on the network functionality. As hub cells are specific subtypes of neurons, it is possible to use *in vivo* cell-specific knockout experiments to explore the behavioral implications of the neural network functional disruption suggested in our work.

## Materials and Methods

### NMC Connectedness Measures

The general connectedness of the NMC was characterized by identifying connected components of the network. A strongly connected component is a group of nodes in which any node is reachable from any other node through a directed path (a series of nodes and directed edges). Intuitively, a strongly connected component reflects a group of recurrently interlinked neurons that could give rise to an anatomical module with functional specialization.

### Small-World Properties

The first property of the small-world analysis is based on the length of the shortest path *l*_*ij*_ between pairs of nodes in the network. A path length between two nodes in the network is expressed as the number of connections along that path. To generalize this property for the entire network, the characteristic path length (*l*) of a network was used, which is the mean shortest path length averaged over all pairs of neurons.


$$l=\frac{1}{n\left(n-1\right)}\sum_{i,j:i\neq j}l_{\text{ij}}$$

Clearly, this measure is well-defined in connected networks where any node is reachable from any other node. The NMC network initially contained a single giant component of 31,329 ± 5 neurons that were mutually reachable (99.95% ± 0.01% of all neurons; see above).

The second property of the small-world analysis is captured by the tendency of nodes to cluster together. The local clustering of individual nodes measures the level at which the neighbors of a node are interconnected among themselves. Let the binary (unweighted) adjacency matrix of a directed network be denoted by *A*; then the local clustering coefficient *c*_*i*_ of a node *i* is defined as,


$$c_{\text{i}}=\frac{{\left(A+A^{T}\right)}_{\text{ii}}^{3}}{2\left(d_{\text{i}}^{\text{tot}}\left(d_{\text{i}}^{\text{tot}}-1\right)-2A_{\text{ii}}^{2}\right)}$$

where $d_{\text{i}}^{\text{tot}}$ depicts the total degree (in-degree + out-degree) of node *i*. Essentially, in directed networks, this definition reflects the ratio of the number of triangles among a node and its neighbors to the number of all possible triangles that could have been formed (Fagiolo, 2007). The value of *c*_*i*_ ranges from 0 (none of the neighbors are connected to each other) to 1 (all neighbors are mutually connected). The network-wide clustering coefficient (*c*) is computed by averaging over all local clustering coefficients.


$$c=\frac{1}{n}\sum_{i}c_{\text{i}}$$

### Hubs Versus the Reference Random Attacks

To selectively attack the highly connected hub neurons we started by ranking all neurons according to their total degree (total number of pre- and post-synaptic cells). Then, we performed several attacks at different strengths (the number of removed hubs). In each attack, given required number of cells to attack (*s*, attack strength), we removed the top degreed cells.

To test the significance of the results observed in hub attacked networks we performed several types of random attacks for comparison. In the first, most naïve random attack, for each hub attack at strength *s* we performed 10 matching random attacks in which randomly selected *s* neurons were attacked. In the second, for each hub attack we counted the number of excitatory *s*_*E*_ and inhibitory *s*_*I*_ neurons that were attacked (*s* = *s*_E_ + *s*_I_); we then performed 10 matching random attacks in which *s*_*E*_ excitatory neurons and *s*_*I*_ inhibitory neurons were selected randomly. We note that a few hub neurons might also be selected, by chance, in these random selection controls.

### Structural Analysis Statistical Tests

To compare the structural disruption of hub attacks to that of random node attacks (Figure 1) we compared the structural metric values (mean path length and clustering coefficient) of the strongest attacks. Specifically, we took the ten strongest hub attacks (*n*_1_ = 10), and the ten matching random attacks for each strength (*n*_2_ = 100). For both metrics (path length or clustering coefficient) a *two-tailed Wilcoxon rank sum test* indicated that the disruption was greater for hub attacks than for matching control (*n*_1_ = 10, *n*_2_ = 100, *P* < 0.001).

For random edge comparisons (Supplementary Figure 1) we took the 100 strongest attacks (*n*_2_ = 100) and compared to the 10 closest hub attacks (*n*_1_ = 10). In agreement with the previous control, also here, a *two-tailed Wilcoxon rank sum test* indicated that the disruption was greater for hub attacks than for matching control (*n*_1_ = 10, *n*_2_ = 100, *P* < 0.001), for both metrics.

### Dense Model of Neocortical Microcircuit (NMC)

Simulations were performed on a previously published model of the NMC of a two-year-old rat of a neocortical microcircuit. Full details on the constructing of the circuits and its simulation methods were described in Markram et al. (2015). The microcircuit (Figure 1B) consisted of 31,346 biophysical Hodgkin-Huxley 3D reconstructed NEURON models with around 7.8 million synaptic connections forming around 36.4 million synapses. Synaptic connectivity between 55 distinct morphological types of neurons (m-types) was predicted algorithmically and constrained by experimental data (Reimann et al., 2015). The densities of ion-channels on morphologically-detailed neuron models were optimized to reproduce the behavior of different electrical neuron types (e-types) and synaptic dynamics recorded *in vitro* (Van Geit et al., 2016). Simulations were run on HPE SGI 8600 supercomputer (BlueBrain 5) using NEURON (Carnevale and Hines, 2006) and CoreNEURON (Kumbhar et al., 2019).

### Simulation of Baseline Spontaneous Activity

To account for the missing long-range connections and missing neuromodulators, neurons were depolarized with a noisy somatic current injection of 100% of first spike threshold (Markram et al., 2015, **Figure 15**). In addition, synapses spontaneous release probability was modified by setting the extracellular calcium concentration [Ca^2+^]_*o*_. Two conditions were tested, [Ca^2+^]_*o*_ of 1.25 and 1.4 mM each positioning the circuit in different activity regimes (Markram et al., 2015, **Figure 15**). Synaptic conductances and kinetics are as in Markram et al. (2015). Each attack was simulated twice with different randomization of the noisy step currents and timing of the spontaneous synaptic release, each time for 10 s.

### Simulation Oscillatory Thalamocortical Input to the NMC

The oscillatory thalamocortical input to the NMC (Figure 4) was generated by following the same principle as in Amsalem et al. (2016) – the spike times of the axons from inhomogeneous Poisson process with time-varying rate as,


$$\lambda \left(\text{t}\right)=B^{*}{\left(\sin\left(t^{*}f^{*}2\pi \right)+1\right)}^{1.5}+b$$

where *t* is time in seconds, *f* the frequency of the oscillatory input was set to 1 Hz, *B*5.83 is a factor, so that the mean firing rate of the oscillatory part would be equal to 7 Hz, and *b* is the baseline spontaneous firing rate which was set to 1 Hz. For Supplementary Figure 1 each simulation was of 10 s, and conducted 5 times with different randomly generated thalamic input.

### Burst Detection

We detected bursts by extracting the multivariate SPIKE-synchronization profile (Mulansky and Kreuz, 2016) for each simulation, smoothing the result using a running mean filter of ∼200 ms and then counting the number of events larger than half the maximal synchronization (but at least larger than 0.15).

### Force-Directed Graph Layout

Visualization of complex networks in an informative and meaningful way is a challenging task. How to position a large number of nodes, densely interconnected with non-obvious organization, in a two-dimensional layout that can expose inherent symmetries and structures such as hubs and clusters?

To provide a layout in which the distance between nodes (cell types) is more or less proportional to their edge weight (connection probability), we employed a Force-directed graph drawing algorithm. The algorithm is based on a physical model that assigns different forces among the nodes. On one hand, to promote attraction between connected nodes spring-like attractive forces, which depend on the distance and edge weight, are simulated. On the other hand, to avoid overlapping of nodes, repulsive forces (such as Coulomb’s law between electrically charged particles) are simulated to separate all pairs of nodes. By iteratively determining all the forces and moving the nodes accordingly, the system gets closer to an equilibrium where all forces add up to zero, and the position of the nodes stays stable.

Here, we used the implementation from D3.js library^1^.

### Visualization

Figures were created using Matplotlib (Hunter, 2007). For analysis we used Python and Numpy (Harris et al., 2020).

## Data Availability Statement

The raw data supporting the conclusions of this article will be made available by the authors, without undue reservation, to any qualified researcher.

## Author Contributions

EG, OA, and IS conceived the study and wrote the manuscript. EG and OA carried out the simulations and the analysis. AS, ML, FS, and HM participated in discussions and helped writing the manuscript. HM and FS developed the *in silico* microcircuit and provided the respective simulation framework and data. All authors contributed to the article and approved the submitted version.

## Conflict of Interest

The authors declare that the research was conducted in the absence of any commercial or financial relationships that could be construed as a potential conflict of interest.

## Publisher’s Note

All claims expressed in this article are solely those of the authors and do not necessarily represent those of their affiliated organizations, or those of the publisher, the editors and the reviewers. Any product that may be evaluated in this article, or claim that may be made by its manufacturer, is not guaranteed or endorsed by the publisher.

## Footnotes

1
https://github.com/d3/d3-force

## Supplementary Material

Supplementary Figure 1 Hub attack disruption is stronger than random attacks with matching number of edges. Same as Figure 1, only the attack strength (*x*-axis) is measured by the number of removed edges.

Supplementary Figure 2 Matched *E*/*I* ratio to hub attack and matched with number of edges **(A–D)** hub attack (black), random attack (orange) and random attack with E/I ratio that is matched to the hub attacks (green) effects on network activity as a function of attack strength. **(A)** Average network firing rate, **(B)** the number of bursts/sec, **(C)** coefficient of variation, and **(D)** global SPIKE-synchronization measure (see section “Materials and Methods” for details about the different attacks and measures). For all measures, hub attack is much more impactful. Panels **(E–H)** same as in Figure 2, only the attack strength (*x*-axis) is measured by the number of removed edges.

Supplementary Figure 3 Effect of hub attacks on the dynamics of the NMC network in the asynchronous regime. **(A)** Raster plot **(top)** and PSTH **(bottom)** of the NMC during spontaneous asynchronous state (see section “Materials and Methods”). **(B,C)** Same as **(A)** after attacking 3,134 and 8,149 hub neurons, respectively. **(D,E)** As in **(B,C)** but for respective random attacks. **(F–G)** Impact of hubs (black) versus random (orange) attacks on network activity as a function of attack strength. **(F)** On the number of bursts/sec, **(G)** on average network firing rate, **(H)** on coefficient of variation, **(I)** on global SPIKE-synchronization measure (see section “Materials and Methods”). For all measures, hub attack is much more impactful.
