# Supplementary material for: The Role of Hub Neurons in Modulating Cortical Dynamics
Source: Front Neural Circuits. 2021 Sep 24;15:718270. doi: 10.3389/fncir.2021.718270 (PMC8500625; doi:10.3389/fncir.2021.718270)
Supplement: Supplementary Figure 1 — Hub attack disruption is stronger than random attacks with matching number of edges. Same as Figure 1, only the attack strength (x-axis) is measured by the number of removed edges. [file Data_Sheet_1.docx]

## Supplementary Material


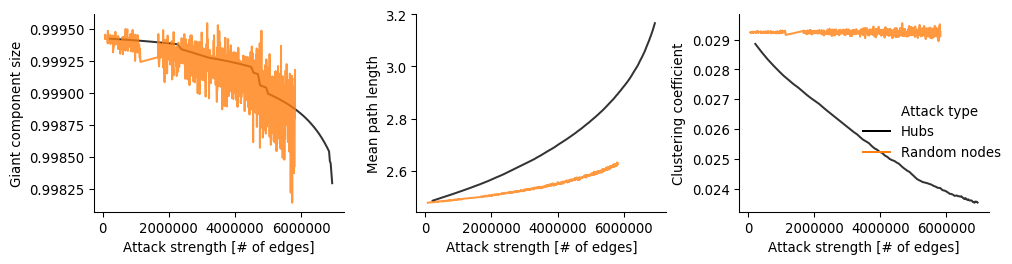


**Supplementary Figure 1: Hub attack disruption is stronger than random attacks with matching number of edges.** Same as Figure 1, only the attack strength (x-axis) is measured by the number of removed edges.


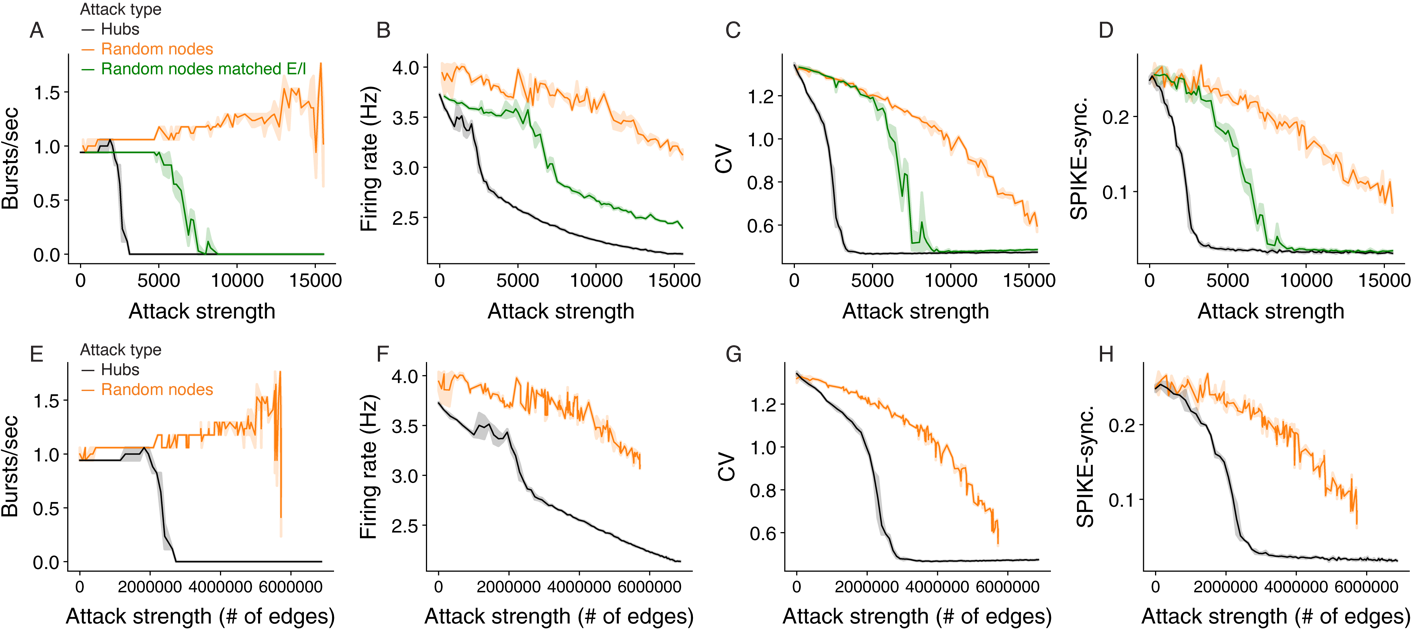


**Supplementary Figure 2. Matched E/I ratio to hub attack and matched with number of edges (A-D)** Hub attack (black), random attack (orange) and random attack with E/I ratio that is matched to the hub attacks (green) effects on network activity as a function of attack strength. **(A)** Average network firing rate, **(B)** The number of bursts/sec, **(C)** Coefficient of variation, and **(D)** Global SPIKE-synchronization measure. (See **Methods** for details about the different attacks and measures). For all measures, hub attack is much more impactful. (**E-H**) same as in Figure 2, only the attack strength (x-axis) is measured by the number of removed edges.


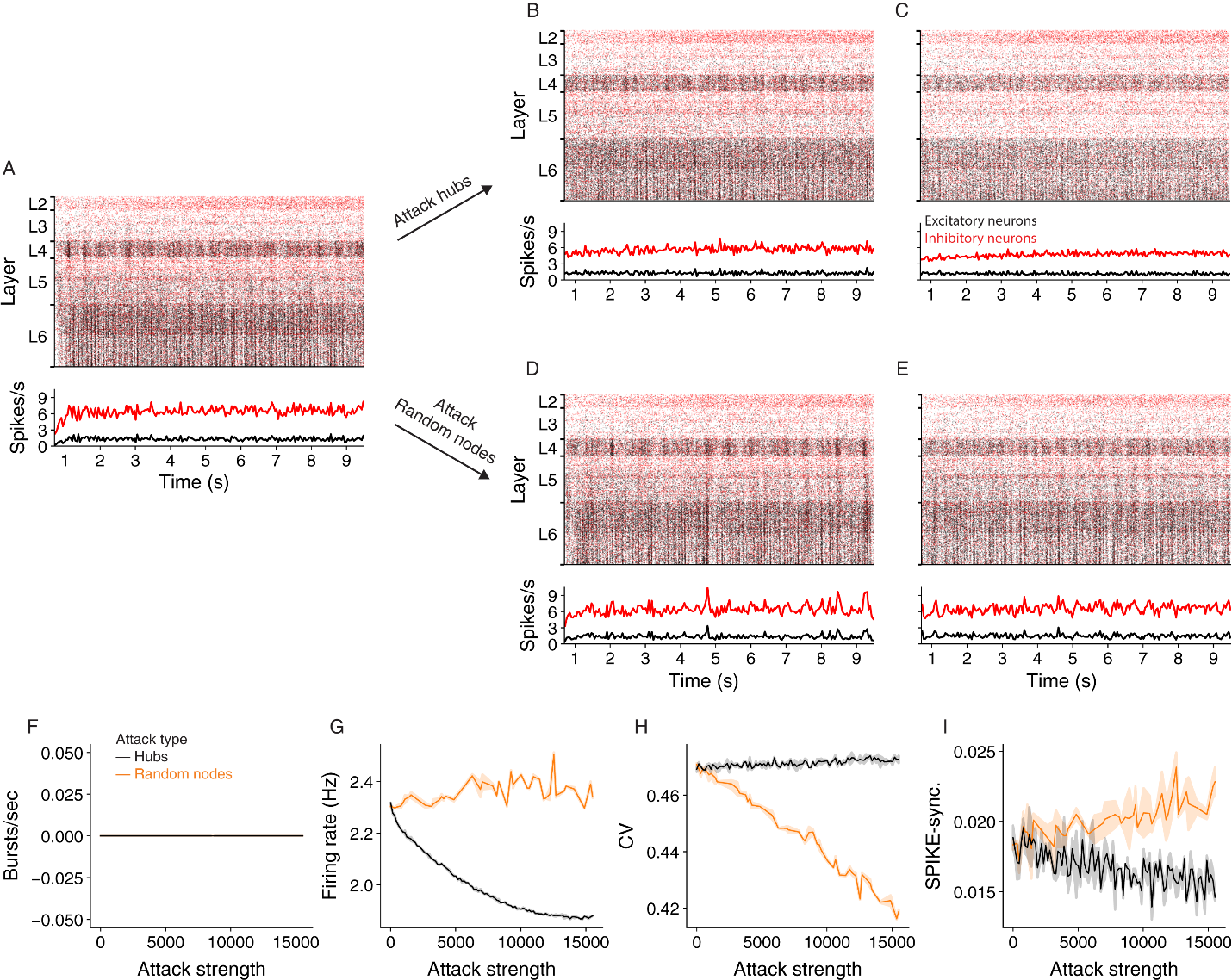


**Supplementary Figure 3. Effect of hub attacks on the dynamics of the NMC network in the asynchronous regime.** **(A)** Raster plot (top) and PSTH (bottom) of the NMC during spontaneous asynchronous state (**Methods**). **(B,C)** Same as (A) after attacking 3,134 and 8,149 hub neurons, respectively. **(D,E)** As in **(B,C)** but for respective random attacks. **(F-G)** Impact of hubs (black) versus random (orange) attacks on network activity as a function of attack strength. **(F)** On the number of bursts/sec, **(G)** On average network firing rate, **(H)** On coefficient of variation **(I)** On global SPIKE-synchronization measure (**Methods**). For all measures, hub attack is much more impactful.
